# RGD4C peptide mediates anti-p21Ras scFv entry into tumor cells and produces an inhibitory effect on the human colon cancer cell line SW480

**DOI:** 10.1186/s12885-021-08056-4

**Published:** 2021-03-25

**Authors:** Chen-Chen Huang, Fang-Rui Liu, Qiang Feng, Xin-Yan Pan, Shu-Ling Song, Ju-Lun Yang

**Affiliations:** 1 School of Medicine, Kunming University of Science and Technology, 727 South Jing Ming Road, Chenggong County, Kunming, 650500 Yunnan Province China; 2 Department of Pathology, 920th Hospital of Joint Logistics Support Force of PLA, 212Daguan Rd, Xishan District, Kunming, 650032 Yunnan Province China

**Keywords:** Ras, Colorectal cancer, RGD4C, Anti-p21Ras scFv

## Abstract

**Background:**

We prepared an anti-p21Ras scFv which could specifically bind with mutant and wild-type p21Ras. However, it cannot penetrate the cell membrane, which prevents it from binding to p21Ras in the cytoplasm. Here, the RGD4C peptide was used to mediate the scFv penetration into tumor cells and produce antitumor effects.

**Methods:**

RGD4C-EGFP and RGD4C-p21Ras-scFv recombinant expression plasmids were constructed to express fusion proteins in *E. coli*, then the fusion proteins were purified with HisPur Ni-NTA. RGD4C-EGFP was used as reporter to test the factors affecting RGD4C penetration into tumor cell. The immunoreactivity of RGD4C-p21Ras-scFv toward p21Ras was identified by ELISA and western blotting. The ability of RGD4C-p21Ras-scFv to penetrate SW480 cells and colocalization with Ras protein was detected by immunocytochemistry and immunofluorescence. The antitumor activity of the RGD4C-p21Ras-scFv was assessed with the MTT, TUNEL, colony formation and cell migration assays. Chloroquine (CQ) was used an endosomal escape enhancing agent to enhance endosomal escape of RGD4C-scFv.

**Results:**

RGD4C-p21Ras-scFv fusion protein were successfully expressed and purified. We found that the RGD4C fusion protein could penetrate into tumor cells, but the tumor cell entry of was time and concentration dependent. Endocytosis inhibitors and a low temperature inhibited RGD4C fusion protein endocytosis into cells. The change of the cell membrane potential did not affect penetrability. RGD4C-p21Ras-scFv could penetrate SW480 cells, effectively inhibit the growth, proliferation and migration of SW480 cells and promote this cells apoptosis. In addition, chloroquine (CQ) could increase endosomal escape and improve antitumor activity of RGD4C-scFv in SW480 cells.

**Conclusion:**

The RGD4C peptide can mediate anti-p21Ras scFv entry into SW480 cells and produce an inhibitory effect, which indicates that RGD4C-p21Ras-scFv may be a potential therapeutic antibody for the treatment of ras-driven cancers.

## Background

Colorectal cancer (CRC) is the third most commonly diagnosed malignancy and one of the leading causes of cancer mortality worldwide and especially in China [1–3]. According to the relevant literature, colorectal cancer accounts for approximately 10% of all annually diagnosed cancers [4, 5]. Although surgery remains the only effective curative option for colorectal cancer [6], 50–60% of tumors have metastasized when diagnosed, thus resulting in metastatic colorectal cancer (mCRC), which is incurable in most cases [7, 8]. In recent years, the application of targeted molecular drugs, such as bevacizumab, cetuximab and panitumumab [9], has led to a significant improvement in the survival rate of patients with metastatic colorectal cancer [10]. However, these targeted drugs also have certain limitations. For example, patients with metastatic colorectal cancer harboring mutations in exon 2 of K-ras did not benefit from anti-epidermal growth factor receptor (EGFR) therapy [11], only those patients with wild-type ras genes could benefit from such treatment [12]. Therefore, ras (K-ras/N-ras) mutation testing is highly recommended in the National Comprehensive Cancer Network (NCCN) guidelines and other guidelines [13–15].

The ras is one of the most commonly mutated genes in all human malignancies, including colon cancer [16–18]. K-ras mutations are present in 22% of tumors, while N-ras and H-ras mutations are less frequent at 8 and 3%, respectively [19, 20]. Ras gene mutations occur in over a third of human colorectal cancer cases [21], and the mutation rate of K-ras is as high as 30–60% [22–25]. Mutated p21Ras proteins become key drivers in the development of cancers [26, 27]. Moreover, wild-type p21Ras overexpression is an important cause of colorectal cancer [28], and the expression rate of p21Ras has been found to reach 29–76% [29]. p21Ras has become a promising therapeutic target for colorectal cancer. However, there is currently no effective and safe treatment to directly target ras-driven neoplasms. Therefore, it is necessary to develop a novel high-efficiency drug that can inhibit mutant p21Ras and overexpressed wild-type p21Ras.

To block the ras signaling pathway and target tumors driven by the ras gene, we previously constructed a single-chain variable fragment antibody (scFv) against p21Ras (anti-p21Ras scFv) that can specifically bind to both mutant p21Ras and wild-type p21Ras [30]. However, the anti-p21Ras scFv can not penetrate the cell membrane, which prevents it from binding to p21Ras in the cytoplasm. As a consequence, it is vital to select an alternative vector to carry the anti-p21Ras scFv into tumor cells to exert antitumor effects.

“Cell-penetrating peptides” (CPPs) are natural or synthetic peptides with the ability to interact with cell membranes to enter cells and/or deliver cargo [31]. Currently, CPPs have been widely used as carriers for delivery of macromolecular drugs, not only enhancing intracellular drug delivery but also improving targeting [32]. RGD, an arginine-glycine-aspartic acid tripeptide, is the interacting site between an integrin and its ligand and shows binding to a variety of integrins [33, 34]. Integrins and RGD-based ligands for integrins are currently being investigated in drug delivery-related areas of research [35, 36]. Alpha(v) beta (3) (αvβ3) is an important integrin. Previous studies revealed that αvβ3 is specifically overexpressed in activated endothelial cells and tumor cells but is not expressed or is rarely expressed in the vast majority of mature endothelial cells and normal cells [37, 38]. Thus, the integrin αvβ3 could become a promising target for cancer therapy. According to these characteristics, several peptides containing RGD sequence-based delivery systems have been designed to specifically bind toαvβ3 receptors. These receptors not only improve targeting potential but also enhance cell membrane internalization to allow therapeutic drugs to enter tumor cells [39, 40]. In this study, to improve the penetration of the anti-p21Ras scFv into tumor tissues via endocytosis, we connected the RGD4C sequence to the N terminus of the anti-p21Ras scFv to construct RGD4C-p21Ras-scFv prokaryotic expression vectors, then the fusion protein RGD4C-p21Ras-scFv (RGD4C-scFv and RGD4C-linker-scFv) was expressed and purified and subsequently investigated the effects of targeting and penetrating the human colorectal cancer cell line SW480 as well as the antitumor effect in vitro.

## Methods

### Cell lines and culture

The human epithelial cell line CACO-2 without mutation of K-ras, human colon cancer cell line SW480 with K-ras mutation [41], the human non-small cell lung cancer cell line A549, human hepatoma cell line Huh7, human glioma cell line U251, normal human lung epithelial cell line BEAS-2B and the normal colonic cell line CCD841 were purchased from the Chinese Academy of Sciences Cell Bank. The cell lines were cultured in DMEM supplemented with 10% fetal bovine serum, 100 U/ml penicillin G, and 100 μg/ml streptomycin under atmospheric conditions of 5% CO_2_ at 37 °C.

### Construction of prokaryotic expression plasmids

The anti-p21Ras scFv was constructed previously in our laboratory [30]. The RGD4C peptide (ACDCRGDCFCG) was developed with phage display technology [42]. The anti-p21Ras scFv gene and RGD4C gene were linked genetically and then inserted into the prokaryotic expression plasmid pET-28a (+) between the *BamH* I and *Hind* III sites. The pET-28a (+) expression vector contains two 6 × His tags to allow immobilized metal ion affinity purification. Recombinant plasmids were sequenced for identification (Qingke, China). Four prokaryotic expression plasmids were constructed: p-scFv, p-RGD4C-scFv, p-RGD4C-linker-scFv, and p-RGD4C-EGFP.

### Expression and purification of fusion proteins

The recombinant expression plasmids were transformed into *Escherichia coli* BL21 (DE3) and selected with kanamycin. After PCR identification, a single positive colony was inoculated into 50 mL of LB medium and grown at 37 °C. The fusion protein was expressed inducibly with 1 mM isopropyl-β-D-thiogalactopyranoside (IPTG) for 5 h at 22 °C. *E. coli* BL21 (DE3) was collected by centrifugation at 12,000 rpm for 20 min and ultrasonicated. The supernatant contained soluble protein, and the precipitate contained inclusion body protein. The soluble recombinant protein and inclusion body protein were collected by bacterial sonication in a bacterial lysis buffer (100 mM sodium chloride, 1 mM EDTA, and 50 mM Tris-HCl buffer, pH 8.0), followed by centrifugation (12,000 rpm, 20 min, 4 °C). The insoluble protein fraction was washed 1 time with inclusion body washing buffer (100 mM sodium chloride, 1 mM EDTA, 1% Triton X-100, 2 M urea, 1 mM dithiothreitol, and 50 mM Tris-HCl, pH 8.0) and then solubilized in a dissolution buffer (8 M urea and 10 mM imidazole in phosphate buffer, pH 7.4). The soluble protein fraction and dissolved inclusion body proteins were purified with the HisPur Ni-NTA Purification Kit (88,229, Thermo, Germany). The purified inclusion body proteins were refolded by gradient dialysis in a dialysis refolding fluid. The expression and purification levels were analyzed by 15%SDS-polyacrylamide gel electrophoresis (SDS-PAGE), and the protein content was determined with the BCA Protein Assay Kit (Thermo Fisher Scientific).

### RGD4C penetration test

#### RGD4C penetrates different tumor cells

RGD4C-EGFP expressed in prokaryotes was used to trace RGD4C penetration of tumor cells. The human tumor cell lines U251, Huh7, SW480 and A549 with high integrin αvβ3 expression and the normal human lung epithelial cell line BEAS-2B were seeded in a 6-well plate at a cell density of 2 × 10^4^, cultured in DMEM overnight and then cultured in DMEM containing RGD4C-EGFP. EGFP fluorescence was observed under an inverted fluorescence microscope.

#### Effect of an endocytosis inhibitor on membrane penetration

SW480 cells were seeded in 6-well plates and cultured overnight. After PBS washing, the endocytosis inhibitor chlorpromazine (50 μM), EIPA (50 μM) or MβCD (1 mM) was added to 300 μl of DMEM containing 10% FBS and co-incubated at 37 °C for 30 min. Then, the cells were incubated with 20 μM RGD4C-EGFP at 37 °C for 5 h. EGFP fluorescence was observed under an inverted fluorescence microscope.

#### Penetration time of RGD4C

SW480 cells were seeded one day in advance and cocultured with 20 μM RGD4C-EGFP at 37 °C in 0.5-h, 1-h, 2-h, and 5-h time gradients. Normal BEAS-2B cells were used as a control group. EGFP fluorescence was observed under an inverted fluorescence microscope.

#### Concentration dependence test

RGD4C-EGFP, at concentrations of 5 μM, 10 μM and 20 μM, was cocultured with previously seeded SW480 cells in 6-well plates for 5 h at 37 °C. EGFP fluorescence was observed under an inverted fluorescence microscope.

#### Temperature-dependent penetration test

SW480 cells were seeded one day in advance. 20 μM RGD4C-EGFP was added to the SW480 cells and incubated at 4 °C or 37 °C for 5 h. EGFP fluorescence was observed under an inverted fluorescence microscope.

#### Effect of ion concentration on membrane penetration

SW480 cells were treated with PBS (K+) in DMEM for 0.5 h, and then were cultured with 20 μM RGD4C-EGFP for 5 h. The control group was treated with PBS to detect the effect of extracellular potential differences on RGD4C peptide penetration. EGFP fluorescence was observed under an inverted fluorescence microscope.

### Detection of the immunoreactivity of RGD4C-p21Ras-scFv

#### Western blot assay

Prokaryotically expressed K-p21Ras [43] was separated by SDS-PAGE, then transferred to polyvinylidene fluoride (PVDF) membranes and incubated with RGD4C-p21Ras-scFv. Next, the PVDF membranes were incubated with anti-Flag tag antibody (Abnova, #2368, China). Subsequently, the membranes were washed and incubated with a goat anti-mouse/rabbit IgG antibody and horseradish peroxidase (HRP) (ZSGB-Bio, ZB-5305, China) at 37 °C for 45 min. After washing with TBST, the protein bands were visualized with a 3,3′-diaminobenzidine (ZSGB-Bio) [29].

#### Elisa

ELISA plates were coated overnight at 4 °C with 5 μg/ml K-p21Ras antigen in 0.05 M carbonate buffer at pH 9.6. The plates were then washed and blocked with 1% bovine serum albumin (BSA)-PBS at 37 °C for 1 h. RGD4C-scFv was diluted 1:100, 1:200, 1:400, 1:800, 1:1600, 1:3200, 1:6400 and 1:12800 with 10% BSA and then allowed to bind to the plates for 1 h at 37 °C. Other control proteins were treated in the same way. After incubation with anti-Flag tag monoclonal antibody (1:1000 dilution) for 1 h at 37 °C, the plates were subsequently incubated with an HRP-conjugated goat anti-mouse/rabbit detection antibody (ZSGB-Bio) (diluted 1:1000 in 10% BSA) for 1 h at 37 °C. Finally, the plates were processed using TMB (3,3′,5,5′-tetramethylbenzidine) peroxidase substrate system (Tiangen Biotechnology, Beijing, China). The absorbance was measured at 570 nm with a microplate reader (Bio-Rad, USA).

### Tumor cell penetration test of RGD4C-p21Ras-scFv

#### Western blot analysis

SW480, Huh7, U251, and A549 tumor cells with high integrin expression and normal BEAS-2B cells without integrin expression were cultured with 20 μM RGD4C-scFv or RGD4C-linker-scFv for 5 h. The cells were lysed in RIPA lysis buffer with a protease inhibitor cocktail containing phenylmethylsulfonylfluoride (PMSF) for 30 min to extract total protein from the tumor cells. Then, the protein electrophoresis was performed with SDS-PAGE gels, and proteins were transferred to polyvinylidene fluoride (PVDF) membranes. β-actin was used as an internal control. Images were converted to the grayscale mode with Photoshop software. Quantification of the target proteins was accomplished by calculating the relative band intensity in the grayscale images of the proteins.

#### Immunocytochemical staining

SW480 cells were cultured with 20 μM anti-p21Ras scFv, RGD4C-scFv, or RGD4C-linker-scFv. Then the cells were fixed in formalin, paraffin-embedded and sectioned. The sections were next exposed to a primary anti-Flag monoclonal antibody (Abnova, #2368, China) and secondary antibody at 1:3000 dilution. The DAB Detection Kit (ZSGB-Bio) was used for staining, and the slides were then counterstained.

#### Immunofluorescence analysis

SW480 cell lines were seeded on coverslips and cultured in dishes at 37 °C with 5% CO_2_, when 80% confluent cells were formed. 20 μM recombinant antibody RGD4C-scFv was added, and incubated for 5 h at 37 °C. And then fixed with 4% paraformaldehyde for 30 min. After permeabilized with PBS containing 0.2% Triton X-100 (Sigma-Aldrich, Darmstadt, Germany) and washed with PBS containing 0.02% Tween-20 (PBST)three-times, the slides were incubated overnight at 4 °C with primary rabbit anti-His Tag mAb (clone number: D3I1O, Cell Signaling TECHNOLOGY, USA) and mouse pan-Ras mAb (clone number: C4, SANTA CRUZ, USA), washed for 5 min with PBS then incubated for 1 h at 37 °C in the dark with FITC-conjugated goat anti-rabbit antibody (ZSGB-BIO) and TRITC-conjugated goat anti-mouse antibody (ZSGB-BIO). Nuclei were stained with 4′,6-diamidino-2-phenylindole (Sigma, Da, Germany) at 25 °C for approximately 15–20 min. The fluorescence signals were analyzed with a fluorescence microscope (OlympusBX51, Tokyo, Japan).

### Antitumor activity of RGD4C-p21Ras-scFv in vitro

#### Cell migration assay

SW480 cells were cultured in 6-well plates to 80% confluence and then starved in serum-free medium overnight. Thereafter, the bottom of the culture plates was scratched with a 200-μl pipette tip. Then, 20 μM anti-p21Ras scFv, RGD4C-scFv, RGD4C-linker-scFv, RGD4C-scFv with 120 μM chloroquine (CQ) and RGD4C-EGFP were added, respectively. CACO-2 and CCD841 cells as the control group were cultured with same way. 20 μM RGD4C-scFv, RGD4C-scFv with 120 μM chloroquine, RGD4C-EGFP were added to the CACO-2 and CCD841 cells, respectively. Cell migration was detected under an inverted microscope (Olympus, Japan) at 0 h, 24 h, and 48 h, and the migration area was calculated using ImageJ software.

#### Colony formation analysis

SW480 cells were cocultured with anti-p21Ras scFv, RGD4C-scFv, RGD4C-linker-scFv, RGD4C-scFv with 120 μM chloroquine and RGD4C-EGFP respectively for 24 h. CACO-2 and CCD841 cells were cocultured with RGD4C-scFv, RGD4C-scFv with 120 μM chloroquine and RGD4C-EGFP, respectively. After digested with 0.25% trypsin and suspended in 10% FBS, the cells were cultured in DMEM containing 10% fetal bovine serum in 6-well plates for 2 weeks at 37 °C with 5% CO_2_. Cell growth was terminated when culture clones could be observed macroscopically. The cells were washed with PBS and fixed with methanol for 15 min. Following 1% Giemsa staining for 10–30 min, the cells were washed with water and dried in air. Colony-forming efficiency was calculated using the formula: colony-forming efficiency = (number of clones/inoculated cell count) × 100%.

#### Cell killing assay

SW480, CACO-2 and CCD841 cells at logarithmic growth phase were inoculated at a density of 1 × 10^4^ cells per well in 96-well plates for 3 days, the anti-p21Ras scFv, RGD4C-scFv, RGD4C-linker-scFv, RGD4C-scFv with 120 μM chloroquine and RGD4C-EGFP were added to SW480 respectively, the RGD4C-scFv, RGD4C-scFv with 120 μM chloroquine, RGD4C-EGFP was added to CACO-2 and CCD841 respectively. At 1, 2, and 3 days, 20 μl of MTT (3-(4,5-dimethylthiazol-2-yl)-2,5-diphenyltetrazolium bromide) (5 mg/ml) was added to each well. After 4 h of incubation with MTT, DMSO (100 μl/well) was added, and the plates were shaken for 10 min. The optical density (OD) value of each well was measured at 490 nm using a microplate reader (Bio-Rad, Model 680).

#### Apoptosis assay

SW480 cells were treated with anti-p21Ras scFv, RGD4C-scFv, RGD4C-linker-scFv, RGD4C-scFv with 120 μM chloroquine and RGD4C-EGFP, respectively, the CACO-2 and CCD841 cells were treated with 20 μM RGD4C-scFv, RGD4C-scFv with 120 μM chloroquine, RGD4C-EGFP respectively for 10 h and then embedded in wax blocks for sectioning. Apoptosis was detected using a terminal deoxynucleotidyl transferase dUTP nick end labeling (TUNEL) assay (In Situ Cell Death Detection Kit; Roche Diagnostics). Nuclei were stained with 4′,6-diamidino-2-phenylindole (DAPI). Apoptotic cells were visualized using a fluorescence microscope.

### Statistical analysis

All data are presented as the mean value ± s.d. Each statistical analysis was performed using SPSS Version 22.0. Comparisons among all groups were performed with a one-way analysis of variance (ANOVA) and the Student–Newman–Keuls method. *P* values < 0.05 was considered statistically significant.

## Results

### Expression and purification of fusion proteins

**Fig. 1:**
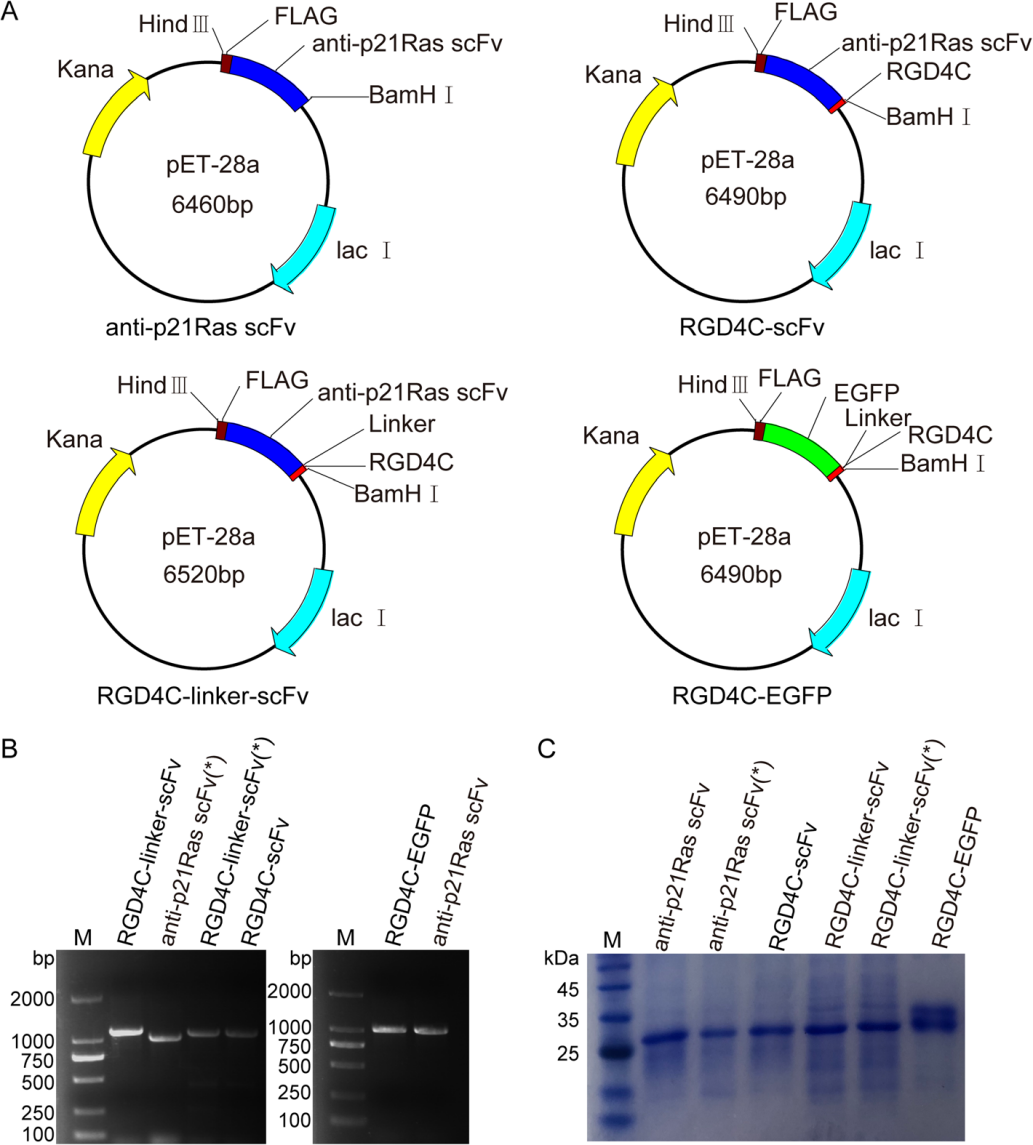
Prokaryotic expression of recombinant proteins. (**a**) The sequences of the anti-p21Ras scFv, RGD4C-scFv, RGD4C-linker-scFv, and RGD4C-EGFP were separately ligated into the pET28a (+) vector to construct recombinant expression plasmids. (**b**) The insertion sequences were detected by polymerase chain reaction (PCR) analysis in *E. coli* BL21 (DE3). (*): unoptimized coding sequence. (**c**) SDS-PAGE analysis showed that the molecular weight of the anti-p21Ras scFv was 34 kDa, that of RGD4C-scFv was 35 kDa, that of RGD4C-linker-scFv was 36 kDa, and that of RGD4C-EGFP was 34 kDa. Codon optimization did not change the molecular weights of the expression products

The construction of prokaryotic recombinant expression plasmids is shown in Fig. 1a. An *E. coli* expression system was used to prepare all the fusion proteins. PCR showed that the recombinant plasmids containing the target gene fragments were successfully transformed into *E. coli* BL21 (DE3) (Fig. 1b). The molecular weight of fusion proteins was determined by SDS-PAGE after purification with nickel metal-affinity resin columns. All of the fusion proteins matched the expected molecular weight, that is, 34 kDa for anti-p21Ras scFv, 35 kDa for RGD4C-scFv, 36 kDa for RGD4C-linker-scFv, and 34 kDa for RGD4C-EGFP. No degradation was observed (Fig. 1c). BCA assay showed that the concentration of purified anti-p21Ras scFv, which was not codon optimized, was 0.96 mg/ml, and that of RGD4C-linker-scFv was 1.06 mg/ml. However, the concentration of codon-optimized anti-p21Ras scFv was 1.41 mg/ml, that of RGD4C-scFv was 1.34 mg/ml, and that of RGD4C-linker-scFv was 1.27 mg/ml. The results revealed that fusion protein expression was higher after codon optimization.

### Influence factors on RGD4C penetration of the tumor cell membrane

**Fig. 2:**
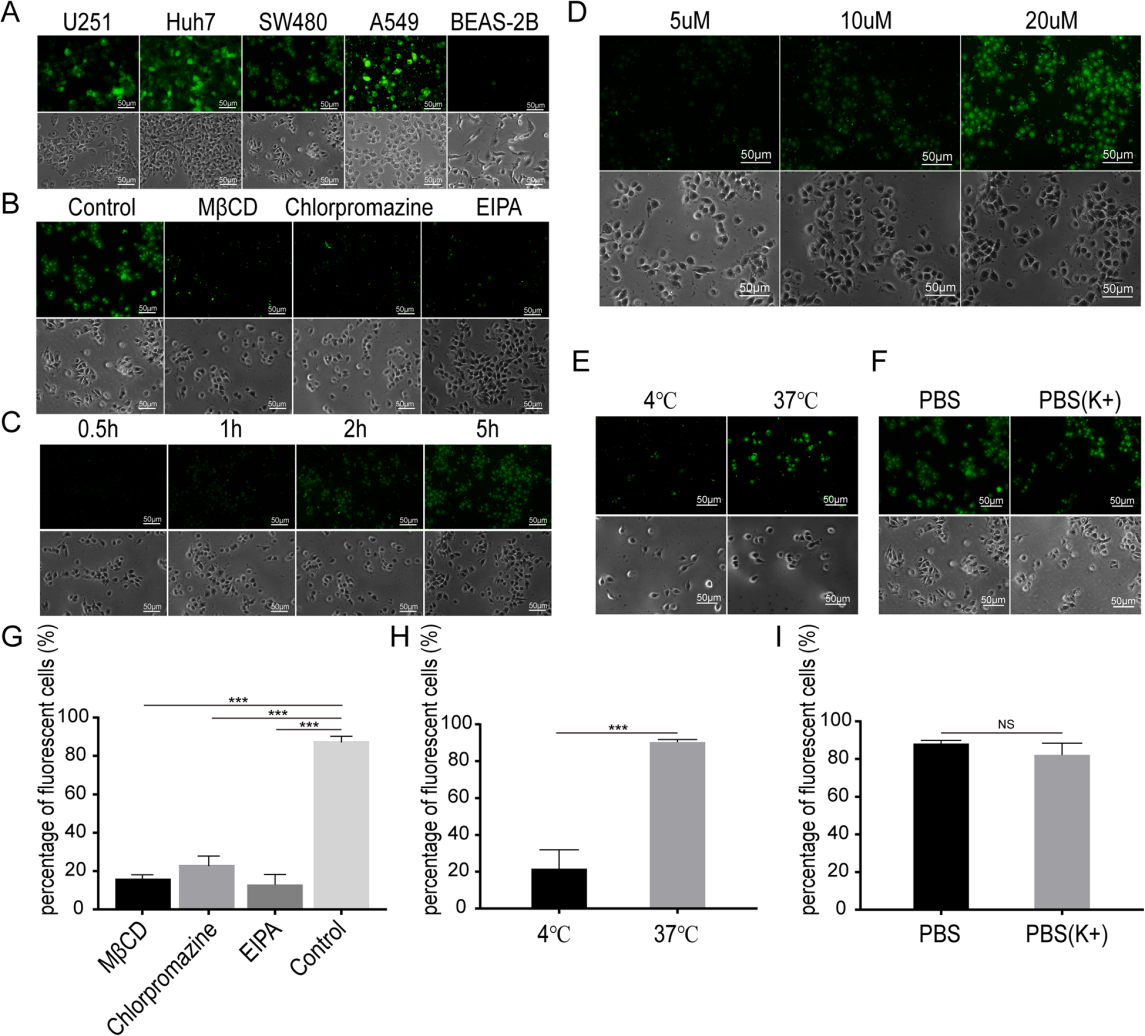
RGD4C penetration test. (**a**) The green fluorescence of EGFP was observed in SW480, U251, Huh7, and A549 tumor cells but not in normal BEAS-2B cells after incubation with RGD4C-EGFP for 5 h. (**b**)(**g**) When the endocytosis inhibitor MβCD, chlorpromazine or EIPA was added to a coculture of RGD4C-EGFP and SW480 cells, the numbers of fluorescent cells decreased significantly compared with the addition of the control reagent (*P* < 0.05). (**c**) SW480 cells were treated with 20 μM RGD4C-EGFP for 0.5 h, 1 h, 2 h or 5 h. Green fluorescence increased with increasing treatment time. (**d**) SW480 cells were treated at 37 °C for 5 h with 5 μM, 10 μM or 20 μM RGD4C-EGFP. The green fluorescence intensity of the SW480 cells increased gradually with increasing RGD4C-EGFP protein concentrations. (**e**) (**h**) The number of fluorescent cells was greater when incubated at 37 °C than when incubated at 4 °C. Incubation at 37 °C caused more RGD4C-EGFP to penetrate SW480 cells than incubation at 4 °C (*p* < 0.05). (**f**) (**i**) PBS and PBS rich in K^+^ were added to separate cocultures of SW480 cells and RGD4C-EGFP for 5 h. There was no significant difference in the percentage of fluorescent cells between the two groups

Fluorescence microscopy was used to observe whether RGD4C-EGFP entered tumor cells with high integrin expression. The green fluorescence signal of RGD4C-EGFP was found in tumor cells, but no fluorescence signal was found in the normal cell line BEAS-2B. These results indicated that RGD4C could penetrate tumor cells with integrin expression but could not penetrate normal cells (Fig. 2a). When different endocytosis inhibitors were added to cocultures of RGD4C-EGFP and SW480 cells, strong fluorescence was observed in the PBS control group, but weak fluorescence was observed in the inhibitor groups (Fig. 2b and g). After RGD4C-EGFP was added to SW480 cells, the green fluorescence signal increased as the culture time increased, and the fluorescence signal was strongest at 5 h (Fig. 2c). When the RGD4C-EGFP concentration was 20 μM, the green fluorescence observed was significantly stronger than that in other experimental groups (Fig. 2d). In an incubation temperature test, stronger green fluorescence was observed at 37 °C, which indicated that the low temperature of 4 °C could inhibit the penetration efficiency of the RGD4C peptide (Fig. 2e and h). Furthermore, the effect of cell membrane potential on the penetration of RGD4C peptide was analyzed and showed that changing cell membrane potential did not affect RGD4C entry into SW480 cells (Fig. 2f and i).

### Immunoreactivity of RGD4C-p21Ras-scFv with p21Ras

**Fig. 3:**
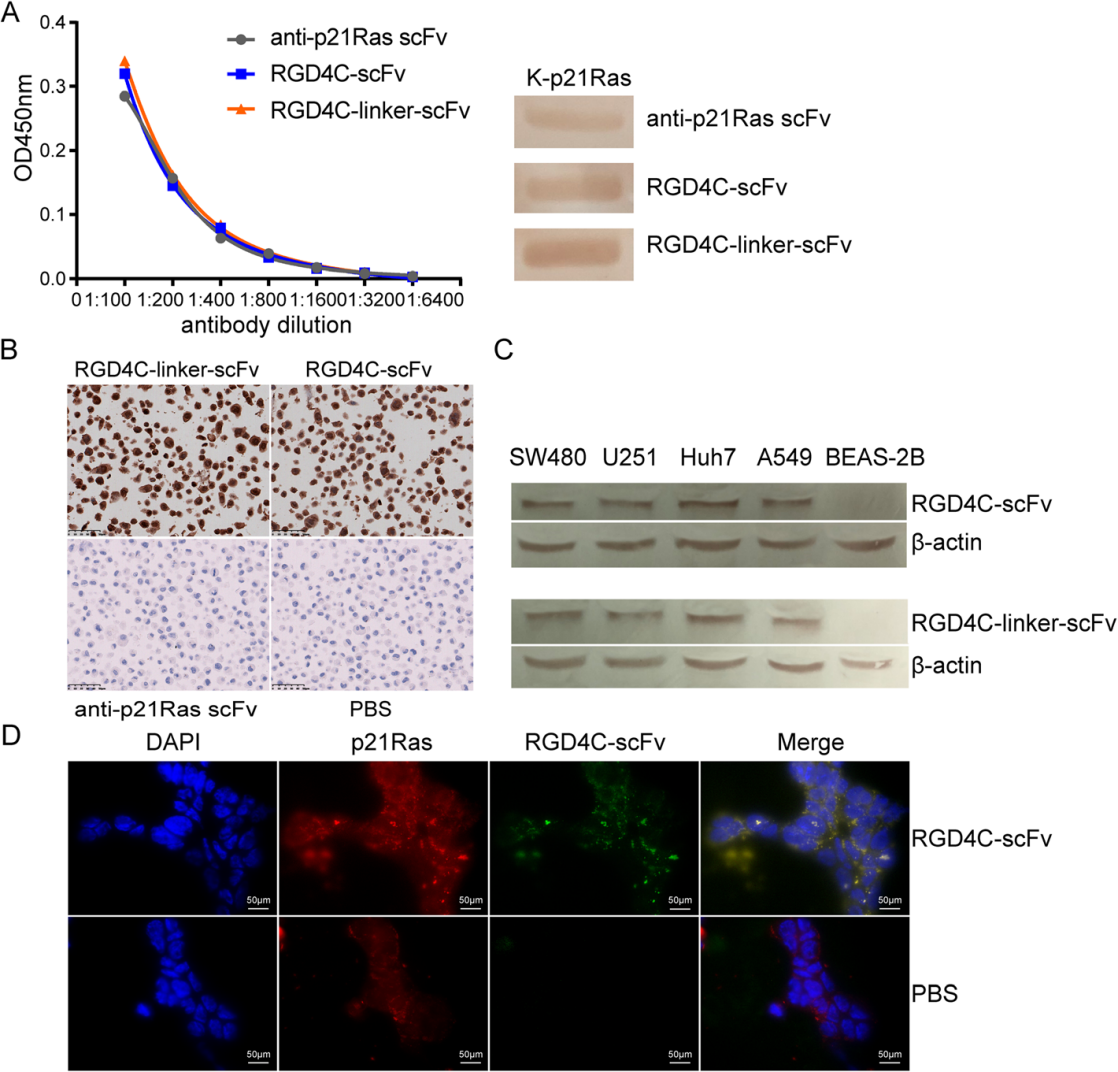
RGD4C-p21Ras-scFv immunoreactivity toward p21Ras and the effect on tumor cell penetration. (**a**) Both ELISA (left) and WB (right) revealed that RGD4C-scFv had almost the same immunoreactivity to p21Ras as the anti-p21Ras scFv and RGD4C-linker-scFv. (**b**) Immunocytochemistry showed that SW480 cells treated with RGD4C-p21Ras-scFv were positively stained, while the anti-p21Ras scFv and PBS incubation groups were negative. We demonstrated that RGD4C-scFv and RGD4C-linker-scFv entered SW480 cells. (**c**) The in vitro tumor targeting of the fusion protein was analyzed by WB. SW480, Huh7, U251, and A549 tumor cells with high integrin expression and normal BEAS-2B cells without integrin expression were cocultured with 20 μM RGD4C-scFv or RGD4C-linker-scFv for 5 h. RGD4C-scFv and RGD4C-linker-scFv were detected in the tumor cells but not in BEAS-2B cells. (**d**) Immunofluorescence detection showed co-localization of the internalized RGD4C-scFv with p21Ras protein in SW480 cells. Red immunofluorescence was observed on the of tumor cells with p21Ras protein, and green immunofluorescence was RGD4C-scFv. Nuclei were counterstained with DAPI (blue)

RGD4C-p21Ras-scFv immunoreactivity was analyzed by western blotting and ELISA to determine whether the RGD4C peptide affects the biological activity of the anti-p21Ras scFv. Western blotting showed that the anti-p21Ras scFv, RGD4C-scFv and RGD4C-linker-scFv could interact with the K-p21Ras antigen, implying that the RGD4C peptide and linker peptide had no effect on the immune activity of the scFv. ELISA results revealed that the binding titers of RGD4C-scFv and RGD4C-linker-scFv for the p21Ras antigen were 1:800, similar to the titer of the anti-p21Ras scFv, which further confirmed that the RGD4C peptide and linker peptide did not affect the titer of the anti-p21Ras scFv (Fig. 3a).

### Ability of RGD4C-p21Ras-scFv to penetrate SW480 cells

Immunocytochemical staining analysis showed that there were high levels of RGD4C-scFv and RGD4C-linker-scFv in SW480 cells, but no positive cells were found in the control of anti-p21Ras scFv and PBS groups (Fig. 3b). Moreover, western blotting revealed that RGD4C-scFv and RGD4C-linker-scFv were detected in tumor cells with high integrin expression, but no fusion proteins were detected in normal cells (Fig. 3c). RGD4C-scFv and RGD4C-linker-scFv had the same targeted penetration ability as RGD4C, and they could penetrate all the tested tumor cell membranes and enter tumor cells. Overall, the results showed that the RGD4C peptide could guide the anti-p21Ras scFv to penetrate tumor cells with high expression of integrin αvβ3. At the same time, the linker did not affect the ability of the RGD4C peptide to carry the scFv into tumor cells. Double color immunofluorescence staining demonstrated that RGD4C-scFv and the p21Ras protein bound together within SW480 cells (Fig. 3d).

### Antitumor effect of RGD4C-p21Ras-scFv in vitro

**Fig. 4:**
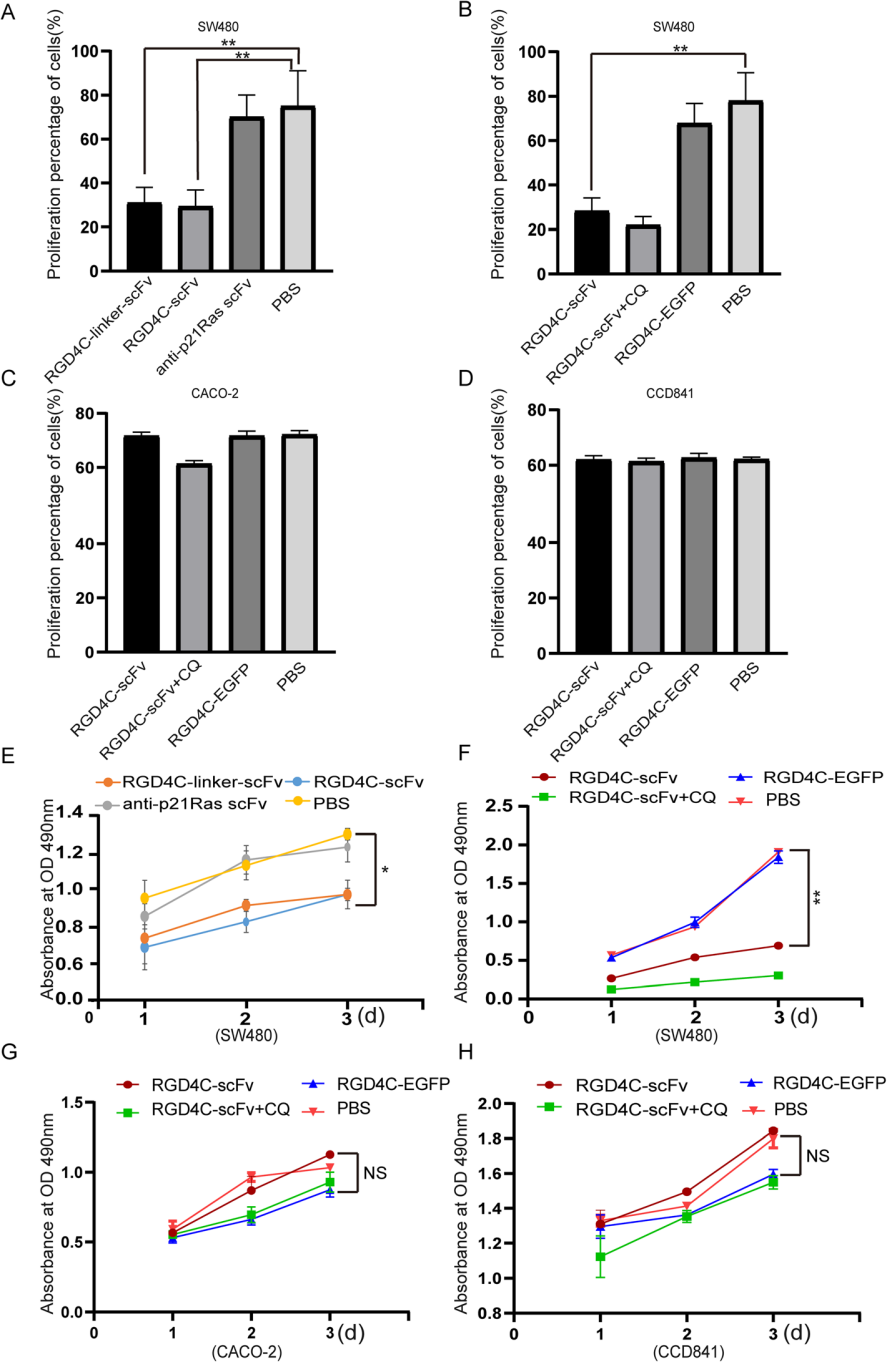
The antitumor efficacy of RGD4C-p21Ras-scFv in vitro. (**a and b**) Cell migration was measured with a scratch test after SW480 cells were cocultured with 20 μM RGD4C-scFv, RGD4C-linker-scFv or the anti-p21Ras scFv for 0 h, 24 h, and 48 h. The migration of SW480 cells was inhibited in the RGD4C-scFv and RGD4C-linker-scFv groups compared with the anti-p21Ras scFv and PBS control groups. (**c and d**) The migration of SW480 cells was inhibited in the RGD4C-scFv and RGD4C-scFv+CQ groups compared with RGD4C-EGFP and PBS groups. Moreover, the migration inhibition effect of RGD4C-scFv+CQ groups was higher than RGD4C-scFv group. (**e-h**) There were no difference the migration of CACO-2 and CCD841 cells in the RGD4C-scFv and RGD4C-scFv+CQ groups compared with RGD4C-EGFP and PBS groups

A scratch test revealed that the area of migrating cells was significantly larger in the anti-p21Ras scFv and PBS treatment groups than in the RGD4C-scFv and RGD4C-linker-scFv treatment groups (Fig. 4a-b), suggesting that RGD4C-scFv and RGD4C-linker-scFv can inhibit the migration of SW480 cells. Moreover, the area of migrating cells was also larger in the PBS and RGD4C-EGFP treatment groups than in the RGD4C-scFv with 120 μM chloroquine and RGD4C-scFv. The migration area of RGD4C-scFv with chloroquine group is still less than in RGD4C-scFv (Fig. 4c-d), suggesting that RGD4C-scFv and RGD4C-linker-scFv can inhibit the migration of SW480 cells, and chloroquine could improve anti-migratory activity in SW480 cells. The inhibitory effect of CACO-2 cells without mutations in K-ras was not obvious (Fig. 4e-f), RGD4C-scFv had no inhibitory effect on normal colonic cell line CCD841(Fig. 4g-h).

**Fig. 5:**
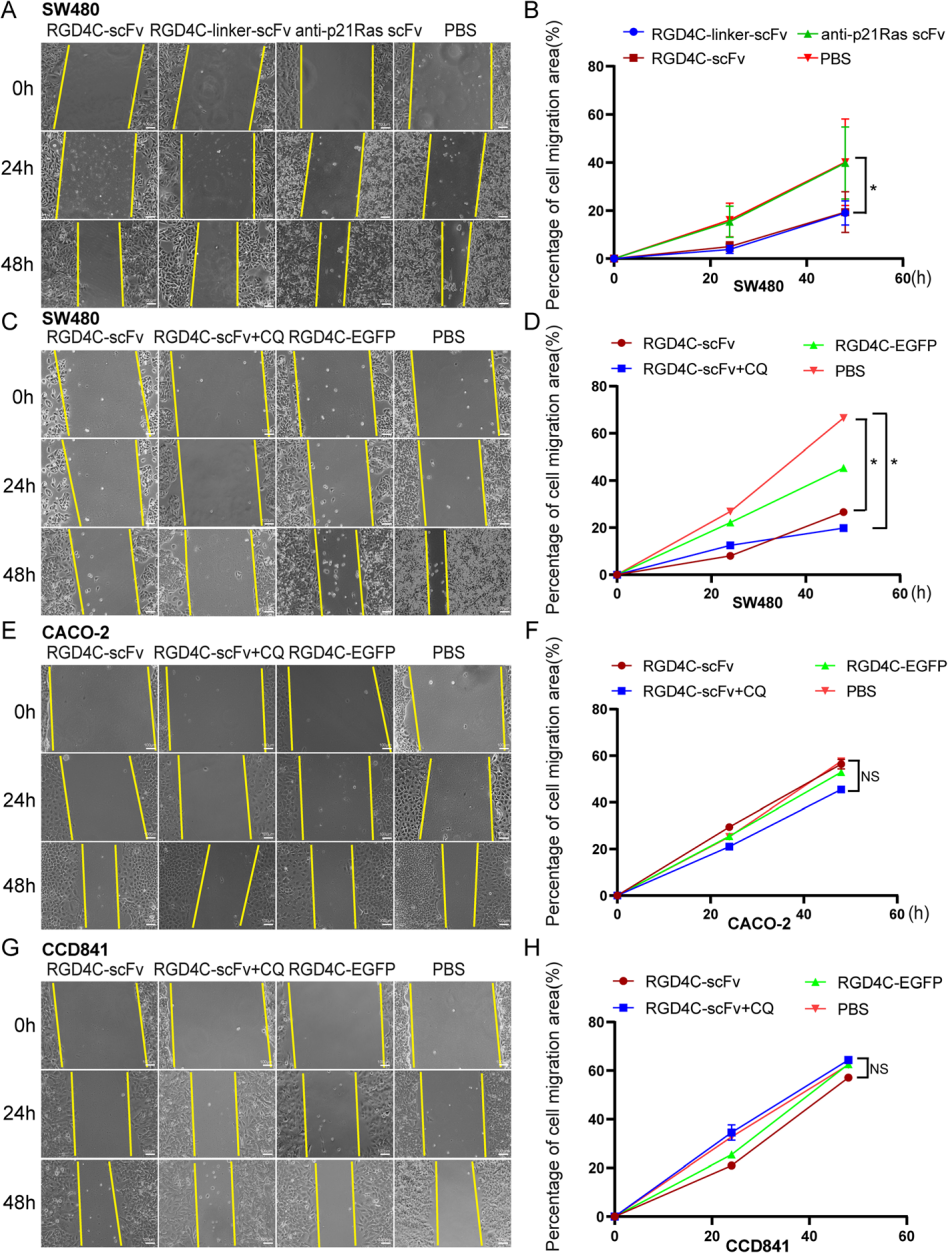
(**a**) A colony formation experiment was performed to detect the effect of RGD4C-scFv on SW480 cell proliferation. SW480 cells were incubated with 20 μM fusion protein. After 2 weeks of incubation, monoclonal cells were stained with Giemsa. The numbers of tumor cell clones in the RGD4C-scFv and RGD4C-linker-scFv groups were significantly lower than those in the anti-p21Ras scFv and PBS groups. (**b**) The clone numbers of SW480 cell in the RGD4C-scFv and RGD4C-scFv+CQ groups were also significantly lower than those in the RGD4C-EGFP and PBS groups. (**c**) However, CACO-2 cell clones had no significant difference between the experimental group and the control group. (**d**) The clone numbers of normal cell CCD841 cells in the RGD4C-scFv and RGD4C-scFv+CQ groups were roughly the same with those in the RGD4C-EGFP and PBS groups. (**e**) After treatment with RGD4C-p21Ras-scFv for 1d, 2 d, or 3 d, the proliferative activity of SW480 cells was tested by an MTT assay. The growth of SW480 cells was inhibited by both RGD4C-scFv and RGD4C-linker-scFv compared with the anti-p21Ras scFv and PBS. (**f**) After treatmented with RGD4C-scFv, RGD4C-EGFP or RGD4C-scFv+CQ for 1d, 2 d, 3 d, the growth of SW480 cells was inhibited by both RGD4C-scFv or RGD4C-scFv+CQ compared with the RGD4C-EGFP and PBS. (**g and h**) After treatment with RGD4C-scFv, RGD4C-EGFP or RGD4C-scFv+CQ for 1d, 2 d, 3 d, neither the RGD4C-EGFP and PBS control groups nor the RGD4C-scFv and RGD4C-scFv+CQ experimental group had any killing effect on the CACO-2 and CCD841 cells

Consistently, the colony formation rates of SW480 cells were 29.58 ± 7.89% in the RGD4C-scFv treatment group and 31.00 ± 7.85% in the RGD4C-linker-scFv group but 70.92 ± 10.42% in the anti-p21Ras scFv group and 75.17 ± 16.50% in the PBS group. This colony formation assay demonstrated that RGD4C-p21Ras-scFv fusion proteins could inhibit the proliferation of SW480 cells (Fig. 5a). We found that the colony formation rates in RGD4C-scFv with chloroquine group was lower than RGD4C-scFv treatment group (Fig. 5b). The control treatment group had no significant effect in CACO-2 and CCD841 cells (Fig. 5c and d).

An MTT assay was performed to evaluate the killing effects of RGD4C-scFv and RGD4C-linker-scFv on SW480 cells. We found that the numbers of live tumor cells in the RGD4C-scFv and RGD4C-linker-scFv groups were lower than those in the anti-p21Ras scFv and PBS groups (Fig. 5e). In addition, the numbers of live tumor cells in the RGD4C-scFv with chloroquine group was also lower than in the RGD4C-scFv group, (Fig. 5f). RGD4C-scFv treatment group had no significant killing effect for CACO-2 cells and CCD841 cells (Fig. 5g and h).

**Fig. 6:**
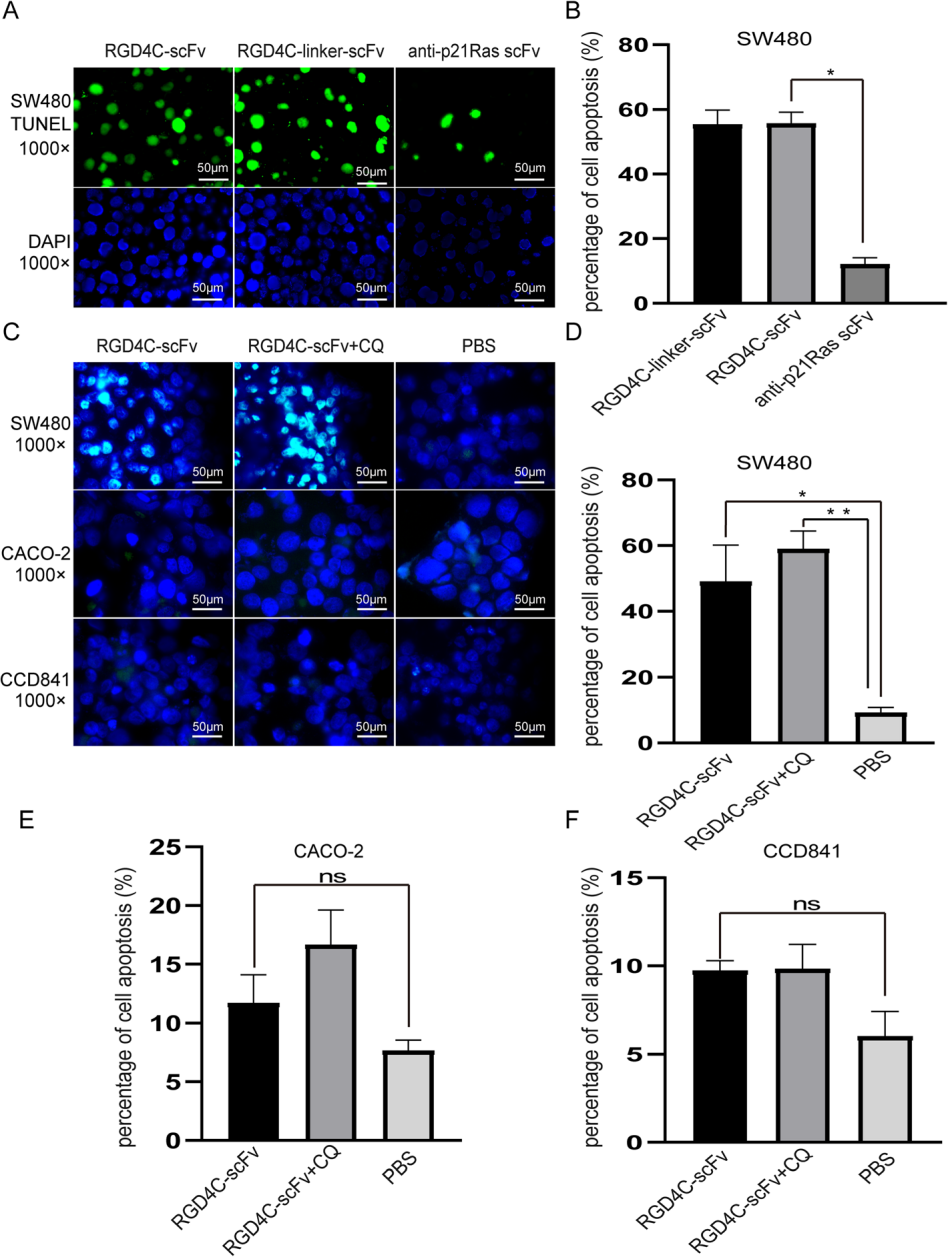
(**a and b**) A TUNEL assay was used to detect SW480 cell apoptosis after treatment with RGD4C-p21Ras-scFv. More apoptotic cells were found in the RGD4C-scFv and RGD4C-linker-scFv groups than in the anti-p21Ras scFv group (green: apoptotic cell, blue: nucleus) (*P* < 0.05). (**c**) There was more green fluorescence were observed in the RGD4C-scFv and RGD4C-scFv+CQ groups of SW480, but other groups showed rarely green fluorescence signal. (**d**) More apoptotic cells were found in RGD4C-scFv+CQ compared with RGD4C-scFv group of SW480. (**e and f**) There were rarely green fluorescence observed with CACO-2 and CCD841 cells no matter RGD4C-scFv, RGD4C-scFv+CQ, RGD4C-EGFP and PBS groups. It implied that RGD4C-scFv did not induce CACO-2 and CCD841 cells apoptosis. Data of groups are presented as the mean ± S.D. *Significantly different from the control group (*P* < 0.05). **P < 0.01 vs controls

TUNEL analysis demonstrated that apoptotic cell numbers increased significantly after treatment with RGD4C-scFv or RGD4C-linker-scFv compared with control treatment. The percentages of apoptotic cellswere54.6 ± 12.1%in the RGD4C-scFv group and 51.6 ± 8.5% in the RGD4C-linker-scFv group. However, in the anti-p21Ras scFv group, the percentage of apoptotic cells was 12.2 ± 2.3%, and there was a significant difference between the RGD4C-scFv and anti-p21Ras scFv groups (*P* < 0.01) (Fig. 6a-b). Meanwhile, the percentages of apoptotic cells in the RGD4C-scFv with chloroquine group was higher than RGD4C-scFv treatment group (Fig. 6c-d). The percentages of apoptotic cells of CACO-2 and CCD841 cells in the RGD4C-scFv with chloroquine group and RGD4C-scFv had little difference compared with PBS group (Fig. 6e-f). It suggested that RGD4C-scFv antibody did not induce apoptosis of tumor cells without K-ras mutation and normal cells. Taken together, the above results indicate that RGD4C can carry the anti-p21Ras scFv into SW480 tumor cells to play antitumor activity.

## Discussion

Assa-Munt N. et al. originally isolated the RGD4C peptide (ACDCRGDCFCG) from a phage-displayed peptide library by screening with the αvβ5 integrin [44]. RGD4C contains the RGD sequence, which can avidly bind to the integrins αvβ3 and αvβ5 but does not bind to other closely related integrins [45]. In addition, the RGD4C peptide can enhance tumor uptake and enable selective delivery of therapeutic or diagnostic agents to tumor sites [46, 47]. In present study, we chose the common cell-penetrating peptide RGD4C as the guiding peptide to carry the anti-p21Ras scFv into tumor cells. First, we constructed a prokaryotic expression system for recombinant RGD4C-p21Ras-scFv fusion proteins. Then, we assessed the factors affecting penetration of the cell membrane by RGD4C and the antitumor activity of the fusion proteins against human colon cancer cells. Additionally, we evaluated whether the linker protein between the RGD4C peptide and anti-p21Ras scFv could influence the biological activity of the RGD4C-p21Ras-scFv fusion protein.

As a guide peptide, the RGD4C peptide has the ability to carry macromolecular drugs through membranes. In recent years, many studies have suggested that the addition of an RGD fragment to peptide drugs may solve the limitation of numerous antitumor drugs being unable to penetrate solid tumors. Natasa Zarovni et al. used RGD4C peptide to carry tumor necrosis factor alpha (TNF), and their experiments showed that the RGD4C peptide successfully increased the uptake of an antibody specific for a tumor-associated antigen and improved the therapeutic properties of the TNF gene [48]. Ebrahim Hosseini et al. reported that modification of interleukin-24 (IL-24) with RGD4C fragments enhanced adherence to tumor cells and improved the anticancer activity of IL-24 [49]. Furthermore, some studies indicated that the RGD peptide is internalized into endosomal compartments by binding to αvβ3 receptors. In this study, we conjugated RGD4C peptide to the anti-p21Ras scFv to improve the ability of the anti-p21Ras scFv to penetrate tumor cells in a targeted manner. Fortunately, our transmembrane experiment showed that the RGD4C peptide included in fluorescent protein conjugates could induce targeted endocytosis to cross the tumor cell membrane, and immunocytochemistry results showed that RGD4C-p21Ras-scFv fusion proteins were able to target and accumulate in SW480 cells because the fusion proteins were powerfully recognized and internalized by integrin αvβ3 receptors expressed on the SW480 tumor cells. Nevertheless, the anti-p21Ras scFv without RGD4C was not detected in SW480 cells, which was consistent with the expected results.

Because the molecular weight of the EGFP protein is similar to that of the anti-p21Ras scFv, we labeled the RGD4C peptide with EGFP to create an RGD-EGFP fusion protein. In the tumor-targeting experiment with the RGD4C peptide, green fluorescence existed in tumor cells with high integrin expression but not in normal cells, which indicated that the RGD4C peptide could cross the cell membrane by recognizing the integrin αvβ3 on the surface of the tumor cells. When changes were made in concentration, temperature, time, endocytosis inhibition or the potential difference, the RGD4C peptide was found to have concentration- and time-dependent membrane penetrating effects. It was found that the penetration ability of the RGD4C peptide weakened after endocytosis inhibitor addition, which indicated that RGD4C function through an endocytosis mode. Moreover, the cell membrane potential did not affect RGD4C entry into tumor cells, suggesting that RGD4C peptide entry into tumor cells occurs via energy-independent endocytosis.

During the construction and expression of fusion proteins, to ensure the activity and function of two interconnected proteins, a specific protein linker may need to maintain the functions of each protein. In our study, we designed two kinds of RGD4C-p21Ras-scFv fusion proteins, one with linker, another without linker. Our data demonstrated that the absence of the linker protein did not affect immunoreactivity or the endocytosis pathway. In addition, there was no significant difference in the antitumor effect between the two fusion proteins. Therefore, we will choose RGD4C-p21Ras-scFv, a simple fusion protein without the linker protein, for subsequent analysis of antitumor efficacy in vivo in future. Future studies are needed to assess the following: (a) the in vivo antitumor activity of RGD4C-p21Ras-scFv fusion proteins; (b) the stability of RGD4C-p21Ras-scFv fusion proteins in the human body; and (c) immunogenicity and toxicity.

## Conclusion

The RGD4C peptide could mediated anti-p21Ras scFv targeting to penetrate tumor cells. RGD4C-p21Ras-scFv fusion proteins could inhibited the migration and proliferation of human colorectal cancer cell line SW480 and induced SW480 cells apoptosis. As a result, these characteristics of RGD4C-p21Ras-scFv fusion proteins could provide the premise for antitumor therapy. It is also suggested that the RGD4C-p21Ras-scFv conjugate may be a novel candidate targeted antitumor drug for ras-driven cancer.

## Data Availability

The data and materials used and analyzed in the current study would be available from the corresponding author on request.

## Abbreviations

CRC: Colorectal cancer
mCRC: metastatic colorectal cancer
EGFR: epidermal growth factor receptor
scFv: single-chain variable fragment antibody
CPPs: Cell-penetrating peptides
HRP: Horseradish peroxidase
IHC: Immunohistochemistry
MTT: 3-(4,5-dimethyl-2-thiazolyl)-2,5-diphenyl-2-H-tetrazolium bromide
PVDF: Polyvinylidene fluoride
