# Correction to: RGD4C peptide mediates anti-p21Ras scFv entry into tumor cells and produces an inhibitory effect on the human colon cancer cell line SW480

**DOI:** 10.1186/s12885-021-08120-z

**Published:** 2021-04-07

**Authors:** Chen-Chen Huang, Fang-Rui Liu, Qiang Feng, Xin-Yan Pan, Shu-Ling Song, Ju-Lun Yang

**Affiliations:** 1 School of Medicine, Kunming University of Science and Technology, 727 South Jing Ming Road, Chenggong County, Kunming, 650500 Yunnan Province China; 2 Department of Pathology, 920th Hospital of Joint Logistics Support Force of PLA, 212Daguan Rd, Xishan District, Kunming, 650032 Yunnan Province China

**Correction to: BMC Cancer 21, 321 (2021)**

**https://doi.org/10.1186/s12885-021-08056-4**

Following publication of the original article [1], the authors reported a typesetting error. Figures 4 and 5 were transposed. The correct Figs. 4 and 5 are supplied below and the original article [1] has been corrected.

**Fig. 4:**
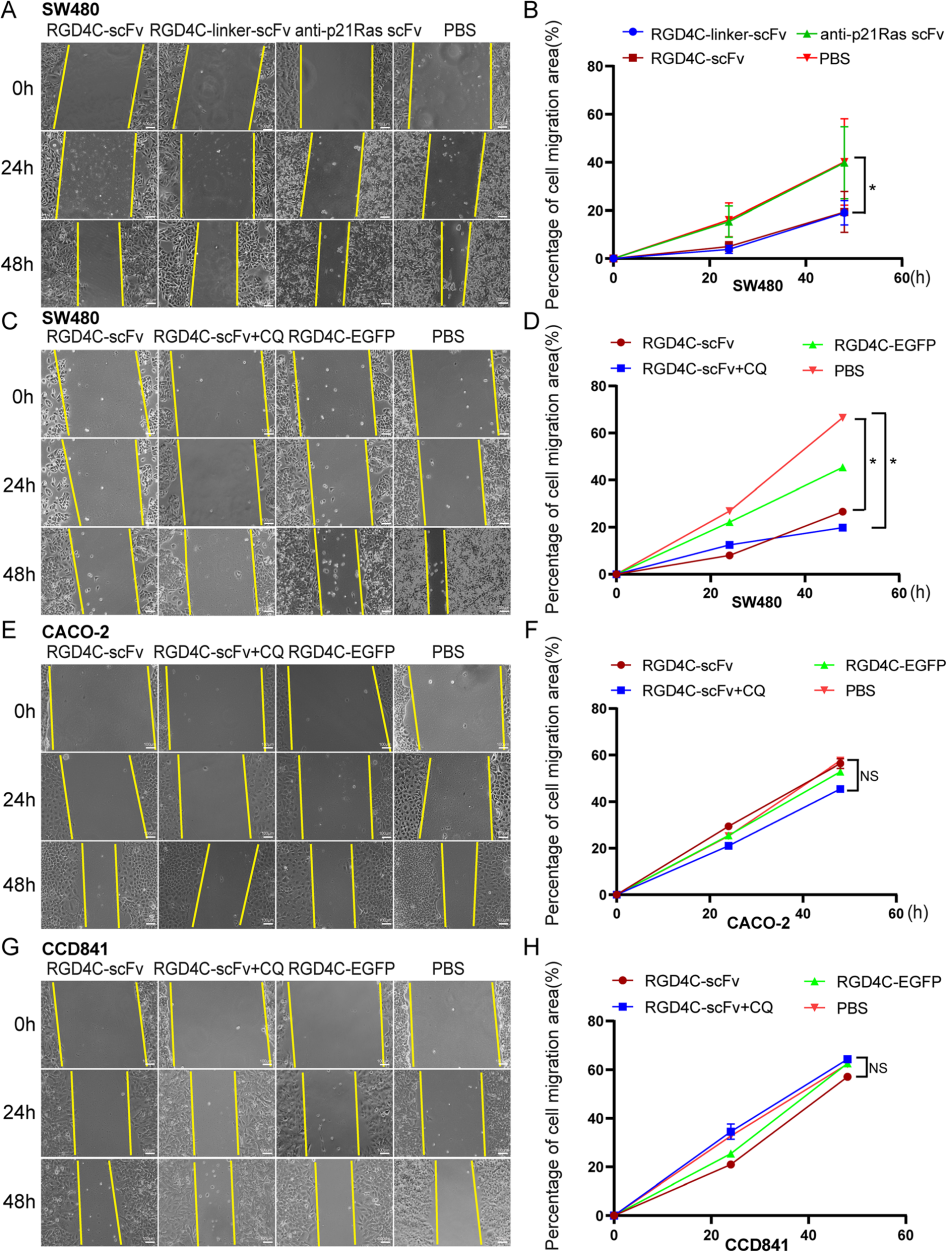
The antitumor efficacy of RGD4C-p21Ras-scFv in vitro. **a** and **b** Cell migration was measured with a scratch test after SW480 cells were cocultured with 20 μM RGD4C-scFv, RGD4C-linker-scFv or the anti-p21Ras scFv for 0 h, 24 h, and 48 h. The migration of SW480 cells was inhibited in the RGD4C-scFv and RGD4C-linker-scFv groups compared with the anti-p21Ras scFv and PBS control groups. **c** and **d** The migration of SW480 cells was inhibited in the RGD4C-scFv and RGD4C-scFv+CQ groups compared with RGD4C-EGFP and PBS groups. Moreover, the migration inhibition effect of RGD4C-scFv+CQ groups was higher than RGD4C-scFv group. **e-h** There were no difference the migration of CACO-2 and CCD841 cells in the RGD4C-scFv and RGD4C-scFv+CQ groups compared with RGD4C-EGFP and PBS groups

**Fig. 5:**
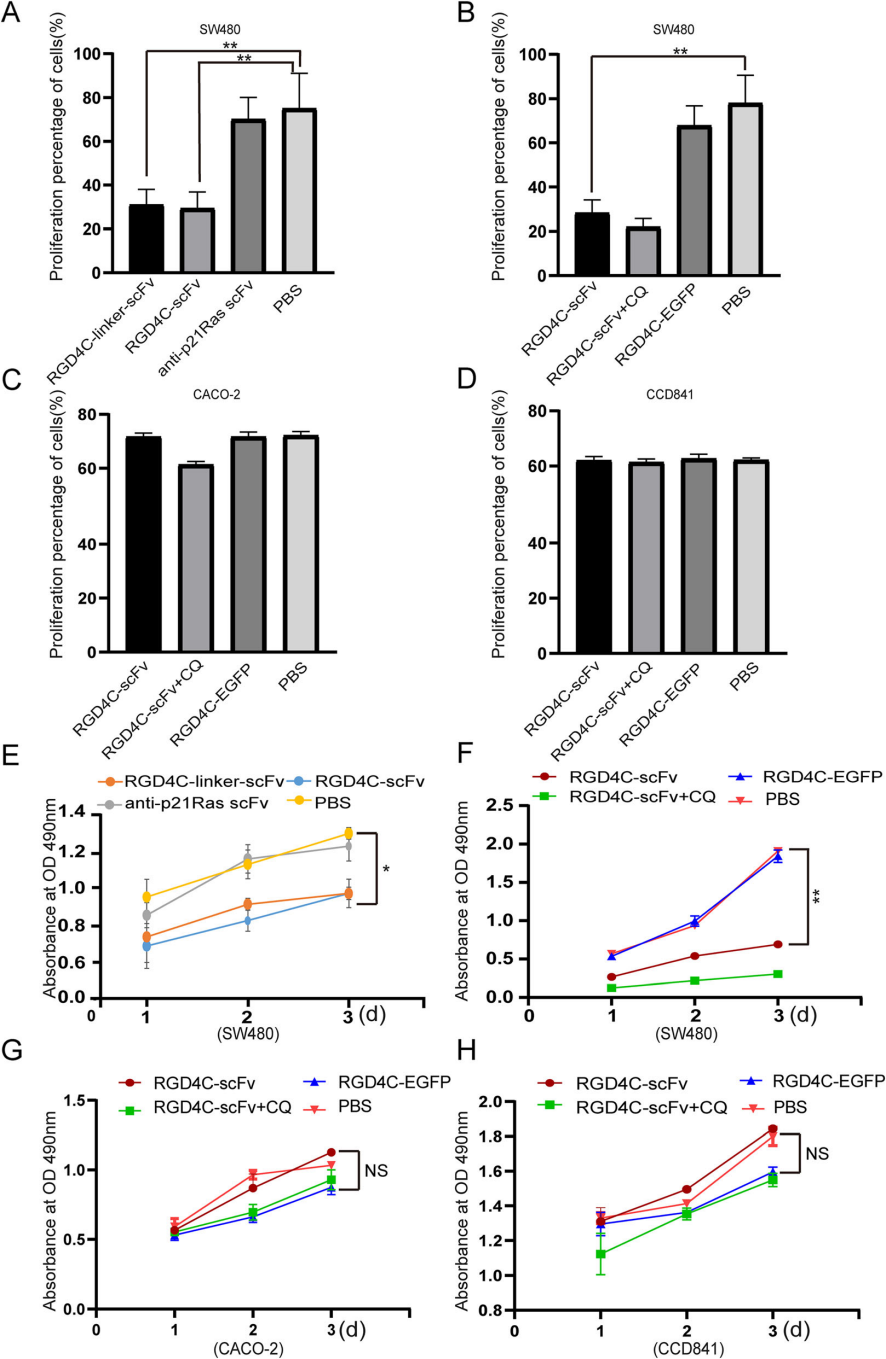
**a** A colony formation experiment was performed to detect the effect of RGD4C-scFv on SW480 cell proliferation. SW480 cells were incubated with 20 μM fusion protein. After 2 weeks of incubation, monoclonal cells were stained with Giemsa. The numbers of tumor cell clones in the RGD4C-scFv and RGD4C-linker-scFv groups were significantly lower than those in the anti-p21Ras scFv and PBS groups. **b** The clone numbers of SW480 cell in the RGD4C-scFv and RGD4C-scFv+CQ groups were also significantly lower than those in the RGD4C-EGFP and PBS groups. **c** However, CACO-2 cell clones had no significant difference between the experimental group and the control group. **d** The clone numbers of normal cell CCD841 cells in the RGD4C-scFv and RGD4C-scFv+CQ groups were roughly the same with those in the RGD4C-EGFP and PBS groups. **e** After treatment with RGD4C-p21Ras-scFv for 1d, 2 d, or 3 d, the proliferative activity of SW480 cells was tested by an MTT assay. The growth of SW480 cells was inhibited by both RGD4C-scFv and RGD4C-linker-scFv compared with the anti-p21Ras scFv and PBS. **f** After treatmented with RGD4C-scFv, RGD4C-EGFP or RGD4C-scFv+CQ for 1d, 2 d, 3 d, the growth of SW480 cells was inhibited by both RGD4C-scFv or RGD4C-scFv+CQ compared with the RGD4C-EGFP and PBS. **g** and **h** After treatment with RGD4C-scFv, RGD4C-EGFP or RGD4C-scFv+CQ for 1d, 2 d, 3 d, neither the RGD4C-EGFP and PBS control groups nor the RGD4C-scFv and RGD4C-scFv+CQ experimental group had any killing effect on the CACO-2 and CCD841 cells
